# Hypercholesterolemia downregulates autophagy in the rat heart

**DOI:** 10.1186/s12944-017-0455-0

**Published:** 2017-03-23

**Authors:** Zoltán Giricz, Gábor Koncsos, Tomáš Rajtík, Zoltán V. Varga, Tamás Baranyai, Csaba Csonka, Adrián Szobi, Adriana Adameová, Roberta A. Gottlieb, Péter Ferdinandy

**Affiliations:** 1 Department of Pharmacology and Pharmacotherapy, Faculty of Medicine, Semmelweis University, Nagyvárad tér 4, H-1089 Budapest, Hungary; 2 Department of Biochemistry, Faculty of Medicine, University of Szeged, Dóm tér 9, H-6720 Szeged, Hungary; 3 Department of Pharmacology and Toxicology, Faculty of Pharmacy, Comenius University, Odbojárov 10, 83232 Bratislava, Slovakia; 4 Heart Institute, Cedars-Sinai Medical Center, 127 S. San Vicente Blvd., Los Angeles, CA 90048 USA; 5 Pharmahungary Group, Szeged, Hungary

**Keywords:** Hypercholesterolemia, Autophagy, Apoptosis, Necroptosis, Programmed necrosis, ATG8, Caspase, Receptor-interacting serine/threonine-protein kinase

## Abstract

**Background:**

We have previously shown that efficiency of ischemic conditioning is diminished in hypercholesterolemia and that autophagy is necessary for cardioprotection. However, it is unknown whether isolated hypercholesterolemia disturbs autophagy or the mammalian target of rapamycin (mTOR) pathways. Therefore, we investigated whether isolated hypercholesterolemia modulates cardiac autophagy-related pathways or programmed cell death mechanisms such as apoptosis and necroptosis in rat heart.

**Methods:**

Male Wistar rats were fed either normal chow (NORM; *n* = 9) or with 2% cholesterol and 0.25% cholic acid-enriched diet (CHOL; *n* = 9) for 12 weeks. CHOL rats exhibited a 41% increase in plasma total cholesterol level over that of NORM rats (4.09 mmol/L vs. 2.89 mmol/L) at the end of diet period. Animals were sacrificed, hearts were excised and briefly washed out. Left ventricles were snap-frozen for determination of markers of autophagy, mTOR pathway, apoptosis, and necroptosis by Western blot.

**Results:**

Isolated hypercholesterolemia was associated with a significant reduction in expression of cardiac autophagy markers such as LC3-II, Beclin-1, Rubicon and RAB7 as compared to controls. Phosphorylation of ribosomal S6, a surrogate marker for mTOR activity, was increased in CHOL samples. Cleaved caspase-3, a marker of apoptosis, increased in CHOL hearts, while no difference in the expression of necroptotic marker RIP1, RIP3 and MLKL was detected between treatments.

**Conclusions:**

This is the first comprehensive analysis of autophagy and programmed cell death pathways of apoptosis and necroptosis in hearts of hypercholesterolemic rats. Our data show that isolated hypercholesterolemia suppresses basal cardiac autophagy and that the decrease in autophagy may be a result of an activated mTOR pathway. Reduced autophagy was accompanied by increased apoptosis, while cardiac necroptosis was not modulated by isolated hypercholesterolemia. Decreased basal autophagy and elevated apoptosis may be responsible for the loss of cardioprotection reported in hypercholesterolemic animals.

## Background

Hyperlipidemias have significant cardiac consequences, since they are among the major risk factors of ischemic heart diseases [1]. The role of atherosclerosis is well studied in these pathologies; however, myocardial effects of hyperlipidemias that do not result in atherosclerosis, such as isolated hypercholesterolemia, is less well understood. Hypercholesterolemia was previously shown to worsen cardiac systolic and diastolic function in cholesterol-fed rabbits [2]. Furthermore, we have previously shown that cholesterol feeding leads to mild contractile dysfunction and cardiac oxidative stress [3]. These data suggest that cardiac metabolism may be affected by hypercholesterolemia well before or even without the development of atherosclerosis. However, it is not well studied which molecular pathways are responsible for the cardiac metabolic effects of hypercholesterolemia.

Autophagy is a ubiquitous cellular housekeeping process [4–6], which is involved in protein quality control and cardiac cytoprotection [7–9]. It is known that autophagy is elevated under nutrient and oxidative stress in ischemia-reperfusion injury [10]. We have previously shown that ischemic preconditioning induces autophagy under normal conditions in adult rat hearts and that autophagy is necessary for cardioprotection by ischemic preconditioning [11]. Similarly, Rohailla et al. showed that remote ischemic preconditioning increases autophagy and decreased the expression of proteins related to the major regulator of autophagy the mammalian target of rapamycin (mTOR) in mice [12]. It is well established that activity of mTOR pathway modulates autophagy [13]. Previous data suggests that mTOR signaling might be elevated while autophagy is diminished in the hearts of hyperlipidemic Yucatan pigs [14], and we have shown that in mice diet-induced obesity results in disturbed cardiac autophagy which is accompanied by increased myocardial injury after ischemia/reperfusion [15]. Cardioprotective effects of ischemic conditioning are attenuated in the presence of cardiovascular risk factors such as aging [16, 17], diabetes [18, 19] and hyperlipidemia [20, 21]. Similarly, our previous studies demonstrated that cardioprotection by various forms of ischemic conditioning is impaired in cholesterol-fed rats [22–25]. However, there is no information whether isolated hypercholesterolemia modulates cardiac autophagy and mTOR pathways in rats.

Insufficient autophagy can promote programmed cell death, apoptosis [26], which results in cardiac damage [27]. Necroptosis (programmed necrosis) is another type of programmed cell death which occurs upon stimulation of death receptors in the absence of caspase-8 activation [28]. Disturbances in cellular energetics, excessive reactive oxygen species production or metabolic changes have been shown to elicit necroptosis and apoptosis [29–32]. It has been shown that apoptosis is upregulated in the hearts of hamsters on hypercholesterolemic diet [30]. Furthermore, Ye et al. showed that TNFα-induced oxidative stress increased necroptosis in parallel with autophagy [33]. Thus, we speculated that isolated hypercholesterolemia may also increase activation of pro-death pathways such as apoptosis and necroptosis in the rat heart.

Therefore, our hypothesis was that (1) cardiac autophagy is diminished in isolated hypercholesterolemia due to increased mTOR signaling, and that (2) pro-death pathways such as apoptosis or necroptosis are upregulated in hypercholesterolemia. Accordingly, the aims of the present study were to investigate the status of autophagy and mTOR pathways in isolated hypercholesterolemia and to examine apoptosis and necroptosis in a hypercholesterolemic rat model.

## Methods

### Animal model

Six-weeks-old male Wistar rats (Crl:WI Strain Code: 003; Charles River Laboratories, Wilmington, MA, USA) were fed with control chow (NORM; *n* = 9) or chow enriched with 2% cholesterol and 0.25% cholic acid (CHOL; *n* = 9) for 12 weeks (Table 1). Animals were allowed to food and water *ad libitum* and chow was changed daily. After the feeding period, body weight of animals were measured and they were anesthetized with diethyl ether and given 500 U/kg heparin i.v. Blood sample (500 μL) was taken from tail vein for further experiments. Hearts were excised and perfused with Krebs-Henseleit buffer according to Langendorff at 37 °C for 10 min as previously described [34]. Hearts were taken and snap-frozen for further biochemical assays.

**Table 1:** Chow composition and nutritional data of chows

	NORM	CHOL
Cholesterol	0.0	2.0
Cholic acid	0.0	0.25
Corn	20.0	20.0
Extracted soybean meal	17.0	17.0
Extracted sunflower seed meal	8.0	8.0
Gluten/corn	4.0	4.0
Protein powder, PL68	1.7	1.7
Wheat middlings	41.7	39.4
Feed lime	1.2	1.2
Calcium phosphate	0.4	0.4
Sodium chloride	0.4	0.4
Zeolite, universal	4.5	4.5
Adhesive	0.6	0.6
Vitamin and trace element supplement	0.5	0.5
Nutritional composition		
Raw protein	22.0	22.0
Raw fat	3.3	3.3
Starch	21.4	20.7
Sugar	5.0	4.9
Raw fiber	6.3	6.3
Raw ash	9.3	9.3
Solids	88.4	88.4
Gross energy (Mj/kg)	14.9	14.5

Data are expressed in % or otherwise noted

### Measurements of glucose and serum lipids

After the 12 week-weeks-long feeding period, glucose, cholesterol and triglyceride levels were measured from plasma of NORM and CHOL rats by colorimetric assays (Diagnosticum, Budapest, Hungary) as previously described [35].

### Measurement of markers of autophagy, mTOR apoptosis and necroptosis by Western blot

Freeze-clamped left ventricles were ground in liquid nitrogen and homogenized with TissueLyser LT (Qiagen, Venlo, The Netherlands) in ice-cold RIPA homogenization buffer (Millipore, Darmstadt, Germany), containing phenylmethylsulfonyl fluoride protease inhibitor (Roche, Basel, Switzerland) and phosphatase inhibitor cocktail (1 mM sodium orthovanadate, 100 mM sodium fluoride and 17.5 mM β-glycero-phosphate; Sigma, St. Louis, MO, USA). Protein concentration was assessed with BCA kit (Thermo Fisher Scientific, Waltham, MA, USA). Protein samples were resolved on 4–20% Criterion TGX gels (Bio-Rad, Hercules, CA, USA) and transferred to Immun-Blot PVDF membranes (Bio-Rad). Equal protein loading was verified with Ponceau staining. Membranes were blocked with 5% nonfat milk (Bio-Rad) or bovine serum albumin (BSA; Santa Cruz Biotechnology, Dallas, TX, USA) or fish skin gelatin (FSG; Sigma) in Tris-buffered saline with 0.05% Tween 20 (TBS-T) for 2 h. Membranes were incubated with primary antibodies in 5% nonfat milk or BSA in TBS-T against late autophagy markers such as LC3 A/B, NBR1 and SQSTM1/p62; early autophagy markers such as Class-III PI3K, Beclin-1 and Rubicon; an autophago-lysosomal marker RAB7; mTOR pathway markers such as phospho-Akt [Thr308], phospho-Akt [Ser473], Akt, phospho-mTOR [Ser2448], mTOR, phospho- ribosomal S6 [Ser235/236], ribosomal S6 (Cell Signaling Technology, Danvers, MA, USA), a modulator of autophagy and mTOR pathway; pro- and anti-apoptotic proteins such as BCL-2, BAX (Sigma) caspase-3 (Novus Biologicals, Littleton, Colorado, USA), necroptosis markers such as RIP1, RIP3 and MLKL (Sigma); and GAPDH (Cell Signaling) or Actin (Sigma) as loading controls. After three washes with TBS-T, secondary antibody conjugated to horseradish peroxidase was added for 1 h at room temperature (in 5% nonfat milk in TBS-T; Cell Signaling). Signals were detected with an enhanced chemiluminescence kit (Bio-Rad) by Chemidoc XRS+ (Bio-Rad) and quantitated in Image Lab 4.1 software (Bio-Rad). Antibodies bound to phospho-epitopes were removed with Pierce Stripping Buffer (Thermo) before incubation with antibodies detecting the total protein.

### Statistical analysis

Values are expressed as mean ± standard error of mean (SEM). Statistical analysis was performed between groups by unpaired two-tailed *t*-test by using GraphPad Prism 5 software. A *p* < 0.05 value was considered significant.

## Results

### Elevated serum cholesterol level in hypercholesterolemic rats

We examined body weight at the 12th week of the diet and found no significant difference between groups (NORM: 551.5 ± 13.0 g vs. CHOL: 529.4 ± 12.2 g; *p* < 0.05). Serum cholesterol, triglyceride and glucose parameters were measured in control and cholesterol-fed rats to assess the state of lipid- and glucose homeostasis. After the feeding period, serum cholesterol level was elevated, although serum triglyceride and glucose levels were unchanged in CHOL group as compared to the NORM group, evidencing isolated hypercholesterolemia in CHOL animals (Table 2). In addition, in a previous study, CHOL animals displayed an altered lipoprotein pattern after 12 weeks of feeding [34].

**Table 2:** Plasma triglycerides, cholesterol, glucose levels in NORM and CHOL groups

	NORM	CHOL
Glucose (mmol/L)	5.32 ± 0.14	5.23 ± 0.09
Cholesterol (mmol/L)	2.89 ± 0.22	4.09 ± 0.62*
Triglycerides (mmol/L)	2.17 ± 0.03	2.26 ± 0.05

Plasma cholesterol level was significantly elevated after 12 weeks of feeding in CHOL animals. NORM (*n* = 9) vs. CHOL (*n* = 9), mean + SEM; *: *p* < 0.05)

### Hypercholesterolemia downregulates autophagy

**Fig. 1:**
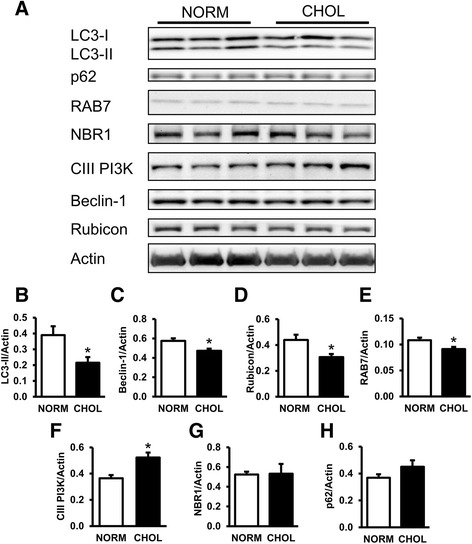
Hypercholesterolemia downregulated cardiac autophagy. **a** Representative Western blots for autophagy-related proteins in the left ventricle of NORM and CHOL rats. **b**-**h** Quantification of LC3-I/II, Beclin-1, Class III PI3K, NBR1, SQSTM1/p62, RAB7 and Rubicon protein expressions as normalized to Actin. NORM (*n* = 4) vs. CHOL (*n* = 5); mean + SEM; *: *p* < 0.05)

In order to establish whether hypercholesterolemia influences autophagy we measured cardiac expression of autophagy markers by Western blot. Hearts of cholesterol-fed animals exhibited a decrease in LC3-II, Beclin-1, Rubicon, and RAB7 (Fig. 1b-e), consistent with downregulation of autophagy initiation and vesicle traffic despite upregulation of the Class-III PI3K involved in autophagy initiation (Fig. 1f). No difference in the expression of markers of autophagic clearance NBR1 and SQSTM1/p62 proteins was detected between groups (Fig. 1g-h). These results suggest that both early and late phases of autophagy were attenuated in the heart of hypercholesterolemic rats.

### Hypercholesterolemia activates mTOR in the heart

**Fig. 2:**
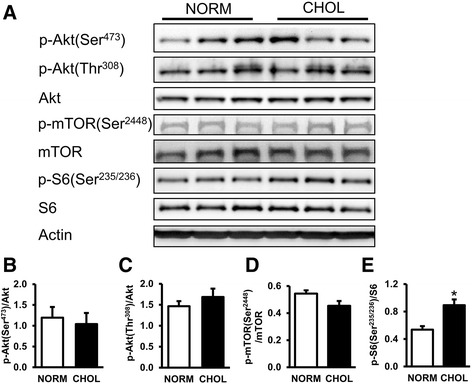
Hypercholesterolemia activated mTOR pathway. **a** Representative Western blots for mTOR pathway-related proteins in the left ventricle of NORM and CHOL rats. **b**-**e** Quantification of phospho-Akt [Ser473; Thr308], phospho-mTOR [Ser2448], and phospho-S6 [Ser235/236] protein expressions as normalized to the corresponding total proteins**.** NORM (*n* = 4) vs. CHOL (*n* = 5); mean + SEM; *: *p* < 0.05)

In order to investigate whether hypercholesterolemia affects mTOR pathway we measured the phosphorylation of mTOR and ribosomal S6 proteins. Our results showed that there was no significant difference in the expressions or phosphorylations of Akt and mTOR protein between groups; however, phosphorylation of ribosomal S6, a surrogate marker of mTOR complex activity, was elevated in CHOL group (Fig. 2b-e). These results indicate that mTOR activity was upregulated in hypercholesterolemia.

### Apoptosis but not necroptosis is elevated in hypercholesterolemia

**Fig. 3:**
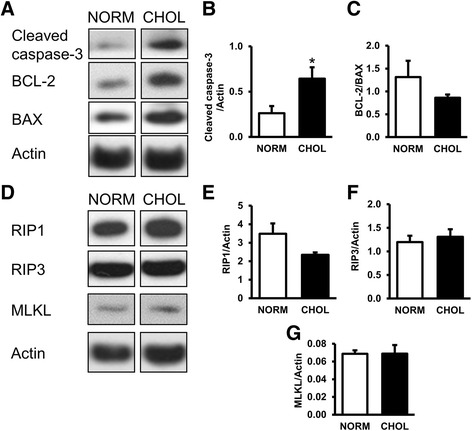
Hypercholesterolemia activated apoptosis, but had no effect on cardiac necroptosis. **a** Representative Western blots for apoptosis-related protein expressions. **b**-**c** Quantification of cleaved caspase-3 expression normalized to Actin and ratio of BCL-2 to BAX. **d** Representative Western blots for necroptosis-related proteins. **e**-**g** Quantification of RIP1, RIP3, MLKL and cleaved caspase-3 as normalized to Actin protein expression. NORM (*n* = 5) vs. CHOL (*n* = 5); mean + SEM; *: *p* < 0.05)

We also investigated whether decreased autophagy in hypercholesterolemia is associated with altered apoptotic and necroptotic pathways. Expression of apoptotic marker cleaved caspase-3 was significantly increased, while BCL-2/BAX protein expression ratio was unchanged (Fig. 3b-c). Furthermore, expression of RIP1, RIP3 and MLKL proteins, major markers of necroptosis, were unchanged (Fig. 3e-g). These results suggest that hypercholesterolemia upregulates apoptosis but not necroptosis in the heart.

## Discussion

**Fig. 4:**
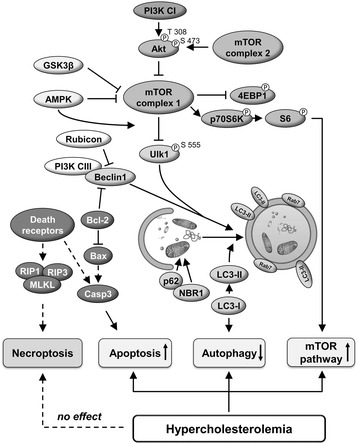
Schematic representation of the cardiac effect of hypercholesterolemia on autophagy, apoptosis, necroptosis and mTOR pathways

This is the first comprehensive analysis of cardiac autophagy and programmed cell death pathways of apoptosis and necroptosis in hypercholesterolemic rats. Here we have shown that hypercholesterolemia induces the attenuation of cardiac autophagy in parallel with the activation of mTOR pathway and an elevation of apoptosis. Moreover, this is the first demonstration that isolated hypercholesterolemia does not influence the expression of major markers of cardiac necroptosis (for schematic representation see Fig. 4).

In this study we showed first that isolated hypercholesterolemia downregulates cardiac autophagy and activates mTOR pathway. Autophagy-related pathways were previously studied in various swine models of mixed hyperlipidemia, which presented somewhat divergent results. Sellke et al. [14] studied swine models of mixed hyperlipidemia where they applied regional cardiac ischemia and assessed autophagy in non-ischemic myocardium. They demonstrated that cardiac mTOR activity was significantly elevated while autophagy marker LC3-II was diminished in hyperlipidemic Yucatan pigs. These findings are in good alignment with our results shown here, however, elsewhere hyperlipidemia decreased expression of Beclin-1, but not of LC3-II in Yorkshire swine [36], while in Ossabaw swine hyperlipidemia elevated cardiac LC3-II and Beclin-1 proteins [37]. Similarly to these findings, the status of cardiac autophagy in other metabolic disorders was also found to be unclear. Recently we showed that diet-induced obesity leads to decreased induction of autophagy in response to fasting in mice involving altered expression of numerous proteins related to metabolic pathways [15], which is in parallel to our current findings. However, in Long-Evans rats fed with high-fat diet we demonstrated only early signs of decreased mitophagy, but no major changes in overall cardiac autophagy or mTOR pathway [38]. In contrast, elsewhere metabolic syndrome induced by high-fat feeding increased cardiac LC3-II and SQSTM1/p62 in mice [39]. Overall, these reports and our current study demonstrate that complex metabolic disorders might modulate cardiac mTOR pathway and autophagy differentially depending on the animal model applied, and they also highlight that focused studies are necessary to decipher the role of specific metabolic pathways on autophagy.

Our results also demonstrated that apoptosis was upregulated by isolated hypercholesterolemia in the rat heart. We assessed an increased cleaved caspase-3 activation, however, the balance of pro- and anti-apoptotic BCL-2 proteins was unaffected by isolated hypercholesterolemia. Similarly, Cheng et al. reported that 8 weeks of cholesterol-enriched diet induced cardiac apoptosis in hamsters, associated with increased expression of BID and BAX [30]. These results correlate well with findings of Zhu et al. who showed that 12 weeks of hypercholesterolemic diet elevated cardiac expression of BAX, decreased BCL-x_L_ but did not affect caspase-3 in swine [40]. Furthermore, Hsu et al. demonstrated that cardiac cleaved PARP was increased in obese C57BL/6 mice on a high fat diet [39]. On the contrary, Osipov et al. showed that hyperlipidemia did not exacerbate cardiac apoptosis after ischemia and reperfusion in a Yucatan swine model based on the findings of decreased cleaved PARP and no difference in activation of caspase-3 [29]. Hypercholesterolemia has also been shown to modulate apoptosis in other tissue types. For example, Perales et al. showed upregulated BAX expression in vascular smooth muscle cells exposed to 25-hydroxycholesterol [41]. Therefore, the majority of these results demonstrates that there is a strong relationship between increased cardiac apoptosis and hypercholesterolemia, although different models of hypercholesterolemia display varying constellations of altered apoptotic markers.

Although several publications report elevated cardiac apoptosis in hypercholesterolemia, data on cardiac necroptosis is scarce. Indeed, this is the first study which assessed the status of necroptosis in vivo in isolated hypercholesterolemia. Here we demonstrate that the expression of major markers of necroptosis are not modulated in the heart of cholesterol-fed rats. However, in a similar model of hypercholesterolemia, cardiac expression of RIP3 mRNA was increased [42], although no other measures of necroptosis were assessed. Nevertheless, these findings demonstrate that the effect of hypercholesterolemia on cardiac necroptosis needs further investigation.

Limitations of this study are that the investigation was performed only on male rats, therefore, we cannot exclude the possibility that sex influences the effect of hypercholesterolemia on cardiac death and survival mechanisms. Furthermore, we did not follow food consumption during the experiments. We did not investigate either the presence of autophagosomes with electron microscopy, or autophagic flux, or the phosphorylation of ribosomal S6 protein through the 90 kDa ribosomal S6 kinase (RSK), which might contribute to the phosphorylation of ribosomal S6 protein independently of the mTOR complex [43]. Another limitation is that the effect of hypercholesterolemia on autophagy, apoptosis, necroptosis and mTOR signaling pathways was assessed only in the left ventricular myocardium.

## Conclusions

In conclusion, this is the first comprehensive analysis of autophagy and programmed cell death pathways in the hearts of hypercholesterolemic rats, demonstrating that autophagy is downregulated, while mTOR pathway and apoptosis are upregulated. This imbalance between pro-survival and death pathways might play a role in the abolition of cardioprotection in hypercholesterolemia. In addition, this is the first demonstration that expression of major markers of cardiac necroptosis is not modulated by isolated hypercholesterolemia.

## Abbreviations

BAX: Apoptosis regulator BAX
BCA: Bicinchoninic acid
BCL-2: Apoptosis regulator Bcl-2
BID: BH3-interacting domain death agonist protein
BSA: Bovine serum albumin
CHOL: Hypercholesterolemic
Class-III PI3K: Class-III phosphoinositide 3-kinase
FSG: Fish skin gelatin
GAPDH: Glyceraldehyde 3-phosphate dehydrogenase
LC3: Microtubule-associated protein light chain 3
MLKL: Mixed lineage kinase domain-like protein
mTOR: Mammalian target of rapamycin
NBR1: Neighbor of BRCA1 gene 1
NORM: Normocholesterolemic
PARP: Poly (ADP-ribose) polymerase
PVDF: Polyvinylidene fluoride
RAB7: Ras-related protein Rab-7a
RIP1: Receptor-interacting serine/threonine-protein kinase 1
RIP3: Receptor-interacting serine/threonine-protein kinase 3
RIPA: Radioimmunoprecipitation assay
RSK: 90 kDa ribosomal S6 kinase
Rubicon: Run domain Beclin-1-interacting and cysteine-rich domain-containing protein
S6: Ribosomal S6 protein
SQSTM1/p62: Sequestosome 1
TBS-T: Tris-buffered saline-Tween 20
