# Erratum to: Hypercholesterolemia downregulates autophagy in the rat heart

**DOI:** 10.1186/s12944-017-0524-4

**Published:** 2017-07-05

**Authors:** Zoltán Giricz, Gábor Koncsos, Tomáš Rajtík, Zoltán V. Varga, Tamás Baranyai, Csaba Csonka, Adrián Szobi, Adriana Adameová, Roberta A. Gottlieb, Péter Ferdinandy

**Affiliations:** 1 Department of Pharmacology and Pharmacotherapy, Faculty of Medicine, Semmelweis University, Nagyvárad tér 4, Budapest, H-1089 Hungary; 2 Department of Biochemistry, Faculty of Medicine, University of Szeged, Dómtér 9, Szeged, H-6720 Hungary; 3 Department of Pharmacology and Toxicology, Faculty of Pharmacy, Comenius University, Odbojárov 10, 83232 Bratislava, Slovakia; 4 Heart Institute, Cedars-Sinai Medical Center, 127 S. San Vicente Blvd, Los Angeles, CA 90048 USA; 5 Pharmahungary Group, Szeged, Hungary

## Erratum

Following publication of the original article [1], it came to the attention of authors that the definition of the term “isolated hypercholesterolemia” used frequently in the article is missing. In this paper, authors define isolated hypercholesterolemia as a hypercholesterolemia without an accompanying hypertriglyceridemia. Therefore, we would like to amend the Background paragraph of the Abstract with the following sentence: “However, it is unknown whether isolated hypercholesterolemia (i.e., hypercholesterolemia without hypertriglyceridemia) disturbs autophagy or the mammalian target of rapamycin (mTOR) pathways.”

In addition, the second sentence of Background section was also incorrectly phrased; which we would like to correct as follows: “The role of atherosclerosis is well studied in these pathologies; however, myocardial effects of hypercholesterolemia is less well understood.”
